# Preoperative COVID-19 and Postoperative Mortality in Cancer Surgery: A South Korean Nationwide Study

**DOI:** 10.1245/s10434-024-15594-1

**Published:** 2024-06-15

**Authors:** Jae-Woo Ju, Soo-Hyuk Yoon, Tak Kyu Oh, Ho-Jin Lee

**Affiliations:** 1 Department of Anesthesiology and Pain Medicine, Seoul National University Hospital, Seoul, Republic of Korea; 2 Department of Anesthesiology and Pain Medicine, Seoul National University College of Medicine, Seoul, Republic of Korea; 3 Department of Anesthesiology and Pain Medicine, Seoul National University Bundang Hospital, Seongnam, Republic of Korea

## Abstract

**Background:**

We evaluated the impact of preoperative COVID-19 on early postoperative mortality in patients undergoing time-sensitive cancer surgery.

**Methods:**

This retrospective, nationwide cohort study included adult patients who underwent various cancer (thyroid, breast, stomach, colorectal, hepatobiliary, genitourinary, lung, and multiple cancer) surgeries under general anesthesia in South Korea in 2022. Patients were grouped according to the duration from the date of COVID-19 confirmation to the date of surgery (0–2 weeks, 3–4 weeks, 5–6 weeks, and ≥7 weeks). Patients without preoperative COVID-19 also were included. Multivariable logistic regression analysis with Firth correction was performed to investigate the association between preoperative COVID-19 and 30-day and 90-day postoperative mortality. The covariates encompassed sociodemographic factors, the type of surgery, and vaccination status in addition to the aforementioned groups.

**Results:**

Of the 99,555 patients analyzed, 30,933 (31.1%) were preoperatively diagnosed with COVID-19. Thirty-day mortality was increased in those who underwent surgery within 0–2 weeks after diagnosis of COVID-19 (adjusted odds ratio [OR], 1.47; 95% confidence interval [CI], 1.02–2.12; *P* = 0.038); beyond 2 weeks, there was no significant increase in mortality. A similar pattern was observed for 90-day mortality. Full vaccination against COVID-19 was associated with reduced 30-day (OR 0.38; 95% CI 0.29–0.50; *P* < 0.001) and 90-day (OR 0.39; 95% CI 0.33–0.46; *P* < 0.001) mortality.

**Conclusions:**

Cancer surgery within 2 weeks of COVID-19 diagnosis was associated with increased early postoperative mortality. These findings support current guidelines that recommend postponing elective surgery for at least 2 weeks after the diagnosis of COVID-19.

**Supplementary Information:**

The online version contains supplementary material available at 10.1245/s10434-024-15594-1.

The coronavirus disease 2019 (COVID-19) pandemic created unprecedented challenges for global healthcare systems and had a significant impact on perioperative management.^1^ Therefore, understanding the effects of preoperative COVID-19 on postoperative outcomes has become a major concern associated with perioperative care.^2^

Early during the pandemic, a multicenter prospective study by the COVIDSurg and GlobalSurg Collaborative highlighted a significant correlation between COVID-19 within 7 weeks before surgery and increased 30-day postoperative mortality.^3^ This finding was pivotal to determining the proper timing for elective surgeries. However, because of its reduced virulence and the advent of vaccines, the impact of preoperative COVID-19 on postoperative outcomes has evolved. Current guidelines implemented in the United States and the United Kingdom recommend postponing elective surgeries for at least 2 weeks after COVID-19 is diagnosed.^4,5^ However, the postponement of elective surgery because of COVID-19 can worsen the prognoses of patients with cancer because of the possibility of cancer progression, thus complicating decision-making.^6,7^ Despite the cancer care guidelines in South Korea during the COVID-19 pandemic advising against delaying surgeries in patients with COVID-19 becaue of the potential risk of cancer progression,^8^ research regarding the optimal timing of cancer surgery for patients with preoperative COVID-19 to minimize the perioperative mortality risk is still lacking. Although the COVID-19 pandemic has ended, the lingering presence of COVID-19 necessitates continued vigilance. Previous studies on the surgical management of cancer in patients with COVID-19 were limited by their small sample sizes and single institutions.^9,10^

Therefore, our study utilized recent national health insurance data from South Korea to investigate the short-term postoperative outcomes of patients with a preoperative diagnosis of COVID-19 who underwent cancer surgery. Our study's findings may aid clinicians in determining the optimal timing for cancer surgery for patients who have been diagnosed with COVID-19.

## Methods

The Institutional Review Board of Seoul National University Hospital exempted the protocol of this study from review because of its retrospective nature and de-identified data. This study was approved by the Korea Disease Control and Prevention Agency (KDCA) and the National Health Insurance Service (NHIS) (approval number KDCA-NHIS-2023-1-366). This study was conducted in accordance with the guidelines of the Declaration of Helsinki and Strengthening the Reporting of Observational Studies in Epidemiology.^11^

### Data Source

All data were obtained from the K-COV-N cohort database, which was created by merging the NHIS and KDCA databases with de-identified registration numbers. Since 2022, the Korean KDCA and NHIS have offered a database known as the K-COV-N cohort database, which combines the NHIS database with COVID-19-related variables to support research on COVID-19.^12^ Similar to other NHIS datasets, this database is anonymized and furnished by the National Health Insurance Corporation. Access for researchers is restricted to designated analysis centers.^13^ The NHIS is a universal mandatory national health insurance system for the Korean population, with an enrollment rate of 97%.^14^ The NHIS database contains records of all inpatient and outpatient medical services, including diagnoses based on *International Classification of Diseases* 10th revision (ICD-10) codes, procedures, and prescription codes.^15^ Additionally, the KDCA database includes detailed information regarding COVID-19 vaccinations in Korea, including the dates of administration and types of vaccinations administered.

### Study Participants

We identified adult patients (age 19 years or older) who underwent cancer surgery in South Korea between January 2022 and December 2022. Surgeries of the thyroid, breast, stomach, lung, liver, gallbladder, pancreas, colorectal, uterus, ovary, kidney, prostate, and testis performed because of cancer were included and defined according to the corresponding ICD-10 codes and procedure insurance claim codes listed in the *Standard Guide to Statistics of Disease and Procedure* published by the Health Insurance Review & Assessment Service in Korea. These codes were used as criteria for generating the surgical healthcare statistics of Korea (Supplemental Table 1).^15^ Multiple cancer surgeries included those involving two or more cancer surgeries performed simultaneously. To ensure a patient-level analysis, we only analyzed the first surgery performed for each patient during the study period. Patients diagnosed with COVID-19 within 30 days after the index cancer surgery, those who underwent emergency surgery, and those who did not receive general anesthesia (procedure codes L0101, L1211, and L1212) for the index cancer surgery were excluded.

**Table 1:** Baseline characteristics and surgical variables

	No preoperative COVID-19 (n = 68,622)	Preoperative COVID-19 infection (by timing of diagnosis before surgery)			
		0–2 weeks (*n* = 3,489)	3–4 weeks (*n* = 2,841)	5–6 weeks (*n* = 2,270)	≥7 weeks (*n* = 22,333)
Age, years					
Mean (SD)	59.7 (13.6)	61.1 (13.9)	58.5 (14.2)	57.8 (14.3)	56.4 (14.3)
19–49	16,114 (23.5)	763 (21.9)	812 (28.6)	700 (30.8)	7,630 (34.2)
50–69	35,161 (51.2)	1,675 (48.0)	1,334 (47.0)	1,046 (46.1)	10,177 (45.6)
≥ 70	17,347 (25.3)	1,051 (30.1)	695 (24.5)	524 (23.1)	4,526 (20.3)
Sex					
Female	43,314 (63.1)	2,152 (61.7)	1,925 (67.8)	1,524 (67.1)	15,781 (70.7)
Male	25,308 (36.9)	1,337 (38.3)	916 (32.2)	746 (32.9)	6,552 (29.3)
Comorbidity					
Congestive heart failure	8468 (12.3)	538 (15.4)	386 (13.6)	306 (13.5)	2,835 (12.7)
Dementia	1,861 (2.7)	143 (4.1)	92 (3.2)	79 (3.5)	687 (3.1)
Chronic pulmonary disease	24,146 (35.2)	1,666 (47.8)	1,357 (47.8)	1,046 (46.1)	10,432 (46.7)
Rheumatologic disease	3,785 (5.5)	195 (5.6)	163 (5.7)	146 (6.4)	1,342 (6.0)
Mild liver disease	32,064 (46.7)	1,929 (55.3)	1,346 (47.4)	1,089 (48.0)	10,654 (47.7)
Diabetes with chronic complications	5,060 (7.4)	301 (8.6)	230 (8.1)	169 (7.4)	1,492 (6.7)
Hemiplegia or paraplegia	451 (0.7)	29 (0.8)	21 (0.7)	19 (0.8)	169 (0.8)
Renal disease	2,746 (4.0)	158 (4.5)	132 (4.6)	99 (4.4)	816 (3.7)
Any malignancy, including leukemia and lymphoma	66,553 (97.0)	3,372 (96.6)	2,744 (96.6)	2,205 (97.1)	21,545 (96.5)
Moderate or severe liver disease	637 (0.9)	63 (1.8)	29 (1.0)	22 (1.0)	160 (0.7)
Metastatic solid tumor	14,009 (20.4)	655 (18.8)	617 (21.7)	459 (20.2)	4,566 (20.4)
AIDS/HIV	86 (0.1)	5 (0.1)	5 (0.2)	8 (0.4)	40 (0.2)
Updated Charlson comorbidity index score					
Median (IQR)	4 (2–8)	5 (3–8)	5 (3–8)	4 (3–8)	4 (3–8)
0–2	17,850 (26.0)	646 (18.5)	603 (21.2)	503 (22.2)	5,021 (22.5)
3–4	19,401 (28.3)	983 (28.2)	810 (28.5)	658 (29.0)	6,573 (29.4)
≥ 5	31,371 (45.7)	1,860 (53.3)	1,428 (50.3)	1,109 (48.9)	10,739 (48.1)
Vaccination					
Not vaccinated	4,044 (5.9)	229 (6.6)	164 (5.8)	132 (5.8)	1,081 (4.8)
Not fully vaccinated	884 (1.3)	29 (0.8)	36 (1.3)	35 (1.5)	270 (1.2)
Fully vaccinated	63,694 (92.8)	3,231 (92.6)	2,641 (93.0)	2,103 (92.6)	20,982 (94.0)
Type of cancer surgery					
Thyroid	14,171 (20.7)	582 (16.7)	654 (23.0)	623 (27.4)	6,023 (27.0)
Breast	17,465 (25.5)	867 (24.8)	780 (27.5)	532 (23.4)	6,398 (28.6)
Stomach	8,318 (12.1)	428 (12.3)	293 (10.3)	202 (8.9)	1,970 (8.8)
Lung	6,417 (9.4)	297 (8.5)	291 (10.2)	250 (11.0)	2,148 (9.6)
Liver	2,545 (3.7)	105 (3.0)	85 (3.0)	74 (3.3)	519 (2.3)
Gallbladder	742 (1.1)	52 (1.5)	23 (0.8)	16 (0.7)	221 (1.0)
Pancreas	1,258 (1.8)	94 (2.7)	55 (1.9)	48 (2.1)	383 (1.7)
Colorectal	11,778 (17.2)	824 (23.6)	435 (15.3)	316 (13.9)	2,665 (11.9)
Uterus	1,715 (2.5)	61 (1.7)	72 (2.5)	61 (2.7)	598 (2.7)
Ovary	1,576 (2.3)	60 (1.7)	64 (2.3)	68 (3.0)	595 (2.7)
Kidney	1,423 (2.1)	51 (1.5)	42 (1.5)	42 (1.9)	400 (1.8)
Prostate	659 (1.0)	33 (0.9)	19 (0.7)	14 (0.6)	222 (1.0)
Testis	150 (0.2)	6 (0.2)	5 (0.2)	7 (0.3)	85 (0.4)
Multiple cancer surgeries	405 (0.6)	29 (0.8)	23 (0.8)	17 (0.7)	106 (0.5)
Income level at the index procedure					
1st quartile (lowest)	19,286 (28.1)	989 (28.3)	827 (29.1)	641 (28.2)	6,248 (28.0)
2nd quartile	18,566 (27.1)	947 (27.1)	751 (26.4)	638 (28.1)	6,454 (28.9)
3rd quartile	15,299 (22.3)	772 (22.1)	624 (22.0)	479 (21.1)	4,793 (21.5)
4th quartile (highest)	15,471 (22.5)	781 (22.4)	639 (22.5)	512 (22.6)	4,838 (21.7)
Area of residence at the index procedure					
Capital city	13,001 (18.9)	620 (17.8)	600 (21.1)	464 (20.4)	4,768 (21.3)
Metropolitan city	17,076 (24.9)	738 (21.2)	725 (25.5)	540 (23.8)	5,748 (25.7)
Other areas	38,545 (56.2)	2,131 (61.1)	1,516 (53.4)	1,266 (55.8)	11,817 (52.9)

Values are expressed as the number (%) unless otherwise indicated

*COVID-19* Coronavirus disease 2019; *SD* standard deviation; *IQR* interquartile range

### Group Classification

The exposure of interest was COVID-19, which was identified using ICD-10 codes (B342, B972, U071, and U072).^16,17^ The date when COVID-19 was confirmed was established as the initial occurrence of the disease associated with specified codes. We stratified patients into the following four groups according to the interval between the confirmed date of preoperative COVID-19 and the index surgery date for cancer: 0 to 2 weeks, 3 to 4 weeks, 5 to 6 weeks, and 7 or more weeks; furthermore, we included a fifth group of patients without a preoperative diagnosis of COVID-19.^3^

### Covariates and Outcomes

We also obtained data regarding baseline characteristics, COVID-19 vaccination-related variables, and cancer surgery types (Table 1). Preoperative comorbidities were defined as at least two relevant ICD-10 codes documented within the year before surgery (Supplemental Table 2). These comorbidities included congestive heart failure, dementia, chronic pulmonary disease, rheumatic disease, mild liver disease, diabetes with chronic complications, hemiplegia or paraplegia, renal disease, any malignancy, including leukemia and lymphoma, moderate or severe liver disease, metastatic solid tumors, and acquired immunodeficiency syndrome/human immunodeficiency virus. The updated Charlson comorbidity index (CCI) score was also calculated.^18^ Regarding the COVID-19 vaccination status, patients were classified as “fully vaccinated” if they received at least one dose of Ad.26.COV2.S or at least two doses of BNT162b2 or mRNA-173 vaccines 14 days or more before the index cancer surgery.^19^ Patients were considered “not fully vaccinated” if they received only one BNT162b2 or mRNA-173 vaccine 14 days or more before the index cancer surgery. The remaining patients were classified as “not vaccinated.” Based on a previous study that demonstrated the beneficial effect of COVID-19 vaccination on individuals without positive COVID-19 test results, we did not consider the temporal order of vaccination or COVID-19 confirmation.^20^ Data regarding income level (as quartiles) and area of residence (capital city, metropolitan city, or other area) were also collected. The primary outcome was 30-day postoperative mortality. The secondary outcome was 90-day postoperative mortality. Because the date of death is included in the K-COV-N cohort database that was utilized during this study, there were no missing data regarding these outcomes.

**Table 2:** Postoperative outcomes after elective cancer surgery

	No preoperative COVID-19 (*n* = 68,622)	Preoperative COVID-19 infection (by timing of diagnosis before surgery)			
		0–2 weeks (*n* = 3,489)	3–4 weeks (*n* = 2,841)	5–6 weeks (*n* = 2,270)	≥7 weeks (*n* = 22,333)
30-day postoperative mortality	326 (0.5)	32 (0.9)	12 (0.4)	14 (0.6)	65 (0.3)
90-day postoperative mortality	928 (1.4)	97 (2.8)	42 (1.5)	36 (1.6)	220 (1.0)

Values are expressed as the number (%)

*COVID-19* Coronavirus disease 2019

### Statistical Analysis

Logistic regression analyses were conducted to compare 30-day postoperative mortality rates across patient groups. To mitigate the bias associated with the low mortality rate presented by our data, we used Firth’s penalized likelihood method, which offers a robust alternative to traditional maximum likelihood logistic regression for analyzing rare events.^21^ To implement this, we employed the FIRTH option in SAS. Univariable logistic regression analyses of the primary outcome were conducted to examine the study groups and potential confounders, including age (19–49 years, 50–69 years, 70 years or older), male sex, updated CCI score (0–2, 3–4, ≥5), COVID-19 vaccination status (fully vaccinated, not fully vaccinated, or not vaccinated), cancer surgery type, income level (as quartiles), and area of residence. Cancer surgery types were categorized as thyroid, breast, stomach, colorectal, hepatobiliary (liver, gallbladder, pancreas), genitourinary (uterus, ovary, kidney, prostate, testis), lung, and multiple. After the univariable analyses, the association between the primary outcome and study group was adjusted for potential confounders in the multivariable logistic regression analysis without applying a variable selection method. The lack of multicollinearity among the variables was established by assessing the variance inflation factor, which was less than 2 before inclusion in the multivariable model. The results are presented as odds ratios (ORs) with 95% confidence intervals (CIs). Logistic regression analyses were repeated in the same manner for the secondary outcome. To confirm our findings, sensitivity analysis was performed by calculating E-value to assess the potential influence of unmeasured confounders on the observed association.^22^

We performed exploratory, prespecified subgroup analyses to determine whether the impact of preoperative COVID-19 varied according to the prespecified subgroups, including age (19–49 years, 50–69 years, and 70 years or older), sex, updated CCI score (0–2, 3–4, ≥ 5), COVID-19 vaccination status, cancer surgery type, income level, and area of residence. For each subgroup, the same multivariable analysis procedure described above was used but with the inclusion of interaction terms. The likelihood ratio test was used to assess the statistical significance of the interaction. Additionally, during the revision process, we conducted another sensitivity analysis by performing logistic regression analyses by using Firth’s penalized likelihood method on the primary and secondary outcomes in abdominopelvic cancer surgeries, which included surgeries for stomach, liver, gallbladder, pancreas, colorectal, uterus, ovary, kidney, prostate, and testis cancers.

Statistical significance was determined as a two-tailed *P*-value of <0.05. Continuous variables are presented as means (standard deviations) or medians (interquartile ranges), as appropriate, and categorical variables are presented as counts (percentages). SAS version 9.4 (SAS Institute, Cary, NC) was used for all analyses.

## Results

**Fig. 1:**
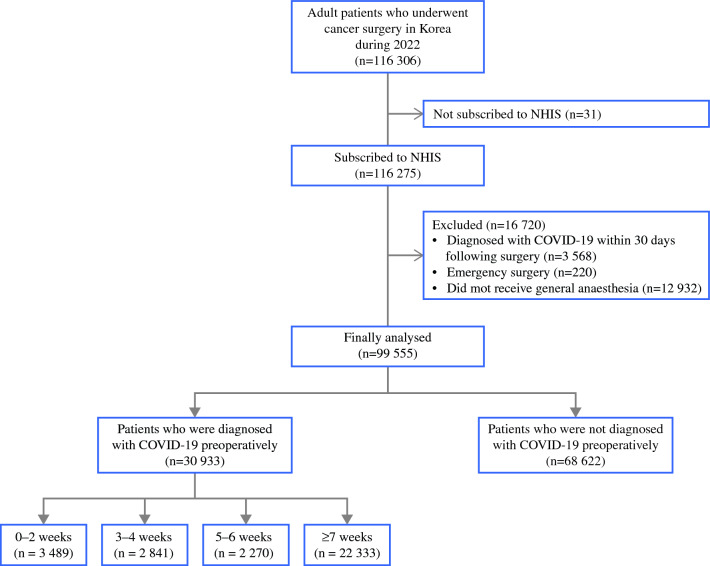
Flowchart of the study. *COVID-19* coronavirus disease 2019; *NHIS* National Health Insurance Service

We obtained data from 116,306 adult patients who underwent cancer surgeries covered by the NHIS in South Korea in 2022. After exclusion, the data of 99,555 patients were analyzed (Fig. 1). Among these patients, 30,933 (31.1%) were preoperatively diagnosed with COVID-19. Patient groups were created according to the time between the COVID-19 diagnosis and the index surgery as follows: within 0 to 2 weeks, 3489 (3.5%) patients; within 3 to 4 weeks, 2841 (2.9%) patients; within 5 to 6 weeks, 2270 (2.3%) patients; and within 7 weeks or more, 22,333 (22.4%) patients.

The 30-day postoperative mortality rate was 0.5% (326/68,622) for patients without preoperative COVID-19 (Table 2). In contrast, the mortality rates of patients with preoperative COVID-19 were 0.9% (32/3489), 0.4% (12/2841), 0.6% (14/2270), and 0.3% (65/22,333) when surgery was performed within 0 to 2 weeks, 3 to 4 weeks, 5 to 6 weeks, and 7 or more weeks after COVID-19 infection, respectively.

Univariate and multivariate logistic regression analyses of the 30-day postoperative mortality rates are presented in Table 3. Patients who underwent surgery within 0 to 2 weeks of the COVID-19 diagnosis had a significantly higher risk of 30-day mortality than those without prior infection (adjusted OR 1.47; 95% CI 1.02–2.12; *P* = 0.038). No significant associations were observed among patients who underwent surgery beyond 2 weeks after the COVID-19 diagnosis. Factors, such as older age, male sex, higher comorbidity index scores (CCI score ≥5), specific cancer surgery types, and lower income levels, were significantly associated with increased 30-day mortality rates. Being fully vaccinated was also significantly associated with a lower 30-day mortality rate (OR 0.38; 95% CI 0.29–0.50; *P* < 0.001). The predictive accuracy of this multivariable model, as indicated by the C-statistic, was 0.860 (95% CI 0.838–0.882).

**Table 3:** Univariable and multivariable logistic regression analyses for 30-day postoperative mortality after elective cancer surgery

	Univariable		Multivariable	
	Unadjusted OR (95% CI)	*P*	Adjusted OR (95% CI)	*P*
Timing of diagnosis of COVID-19 before surgery				
No preoperative COVID-19	Reference		Reference	
0–2 weeks	1.97 (1.37–2.82)	< 0.001	1.47 (1.02–2.12)	0.038
3–4 weeks	0.92 (0.52–1.63)	0.785	0.90 (0.51–1.59)	0.731
5–6 weeks	1.34 (0.79–2.28)	0.272	1.50 (0.88–2.54)	0.134
≥7 weeks	0.62 (0.47–0.80)	< 0.001	0.78 (0.59–1.01)	0.059
Age, years				
0–49	Reference		Reference	
50–69	4.14 (2.48–6.92)	< 0.001	1.73 (1.03–2.92)	0.039
≥ 70	20.13 (12.26–33.05)	< 0.001	5.30 (3.16–8.87)	< 0.001
Male (vs. female)	3.75 (3.08–4.57)	< 0.001	1.59 (1.30–1.95)	< 0.001
Updated Charlson comorbidity index				
0–3	Reference		Reference	
4–5	2.01 (1.31–3.10)	0.002	1.27 (0.83–1.96)	0.272
≥ 6	6.37 (4.37–9.27)	< 0.001	2.72 (1.86–3.97)	<0.001
Fully vaccinated (vs. not vaccinated or not fully vaccinated)	0.43 (0.33–0.56)	< 0.001	0.38 (0.29–0.50)	< 0.001
Type of cancer surgery				
Thyroid	Reference		Reference	
Breast	0.55 (0.22–1.39)	0.207	0.52 (0.21–1.29)	0.156
Stomach	11.78 (6.31–22.01)	< 0.001	4.50 (2.36–8.56)	< 0.001
Colorectal	25.52 (14.09–46.22)	< 0.001	8.33 (4.51–15.40)	< 0.001
Hepatobiliary	22.92 (12.30–42.69)	< 0.001	8.18 (4.31–15.52)	< 0.001
Genitourinary	6.11 (3.04–12.26)	< 0.001	3.32 (1.64–6.69)	0.001
Lung	10.35 (5.45–19.64)	< 0.001	3.73 (1.93–7.21)	< 0.001
Multiple cancer surgeries	18.32 (6.61–50.80)	< 0.001	6.38 (2.28–17.87)	< 0.001
Income level at the index procedure				
1st quartile (lowest)	Reference		Reference	
2nd quartile	0.43 (0.33–0.57)	< 0.001	0.54 (0.41–0.70)	< 0.001
3rd quartile	0.71 (0.56–0.91)	0.007	0.78 (0.61–1.00)	0.048
4th quartile (highest)	0.70 (0.54–0.89)	0.004	0.64 (0.50–0.82)	< 0.001
Residence level at the index procedure				
Capital city	Reference		Reference	
Metropolitan city	0.92 (0.68–1.25)	0.614	1.01 (0.74–1.36)	0.963
Other area	1.28 (1.00–1.65)	0.055	1.11 (0.87–1.43)	0.399

*COVID-19*, coronavirus disease 2019; *OR*, odds ratio; *CI*, confidence interval

In the sensitivity analysis for the 30-day postoperative mortality, the E-value calculated for the initially observed result where patients who underwent surgery within 0 to 2 weeks of COVID-19 diagnosis had a significantly higher risk compared with those without COVID-19 infection was 1.16. In other words, if there were an unmeasured confounder with an OR greater than 1.16, the risk within the 0 to 2 weeks interval would not be significantly different from that of patients without preoperative COVID-19, suggesting that this finding does not appear to be robust against potential unmeasured confounders. Conversely, to alter the nonsignificant findings to significant ones for patients who underwent surgery 3 to 4 weeks, 5 to 6 weeks, and 7 or more weeks after COVID-19 infection, the E-values required to shift the OR to 1.1 and its lower 95% CI to 1.01 were calculated as 3.37, 2.07, and 2.82, respectively. These findings indicate that nonsignificant findings in patients who underwent surgery more than 2 weeks post-COVID-19 infection are moderately robust against potential unmeasured confounding.

Univariable and multivariable logistic regression analyses of the 90-day postoperative mortality rates are presented in Table 4. The multivariable logistic regression analysis revealed that surgery within 0 to 2 weeks of COVID-19 was significantly associated with an increased 90-day mortality rate compared to that of individuals without COVID-19 (adjusted OR 1.60; 95% CI 1.29–1.99; *P* < 0.001). Surgeries performed more than 2 weeks after COVID-19 was diagnosed were not significantly associated with the 90-day mortality rate. Being fully vaccinated was correlated with a lower risk of 90-day mortality (OR 0.39; 95% CI 0.33–0.46; *P* < 0.001). The predictive accuracy of this model, as indicated by the C-statistic, was 0.855 (95% CI 0.842–0.868). For the 90-day postoperative mortality, the E-values for the significance of each group were 1.90 (0–2 weeks), 1.88 (3–4 weeks), 1.67 (5–6 weeks), and 1.91 (7 or more weeks), respectively.

**Table 4:** Univariable and multivariable logistic regression analyses for 90-day postoperative mortality after elective cancer surgery

	Univariable		Multivariable	
	Unadjusted OR (95% CI)	*P*	Adjusted OR (95% CI)	*P*
Timing of diagnosis of COVID-19 before surgery				
No preoperative COVID-19	Reference		Reference	
0–2 weeks	2.10 (1.70–2.59)	<0.001	1.60 (1.29–1.99)	< 0.001
3–4 weeks	1.11 (0.81–1.51)	0.521	1.09 (0.79–1.50)	0.594
5–6 weeks	1.19 (0.85–1.66)	0.304	1.31 (0.93–1.84)	0.122
≥ 7 weeks	0.73 (0.63–0.84)	< 0.001	0.91 (0.78–1.06)	0.211
Age, years				
0–49	Reference		Reference	
50–69	4.01 (3.02–5.32)	< 0.001	1.61 (1.21–2.16)	0.001
≥ 70	17.54 (13.33–23.08)	< 0.001	4.29 (3.21–5.73)	< 0.001
Male (vs. female)	3.47 (3.10–3.89)	< 0.001	1.47 (1.31–1.66)	< 0.001
Updated Charlson comorbidity index				
0–3	Reference		Reference	
4–5	1.68 (1.29–2.19)	< 0.001	1.09 (0.84–1.42)	0.516
≥ 6	6.88 (5.51–8.59)	< 0.001	3.15 (2.52–3.95)	< 0.001
Fully vaccinated (vs. not vaccinated or not fully vaccinated)	0.44 (0.38–0.51)	< 0.001	0.39 (0.33–0.46)	< 0.001
Type of cancer surgery				
Thyroid	Reference		Reference	
Breast	0.85 (0.49–1.45)	0.546	0.79 (0.46–1.35)	0.384
Stomach	16.67 (11.14–24.96)	< 0.001	7.27 (4.79–11.03)	< 0.001
Colorectal	33.21 (22.50–49.02)	< 0.001	12.25 (8.19–18.32)	< 0.001
Hepatobiliary	27.68 (18.44–41.54)	< 0.001	11.05 (7.27–16.79)	< 0.001
Genitourinary	8.21 (5.28–12.76)	< 0.001	4.78 (3.06–7.46)	< 0.001
Lung	12.42 (8.19–18.84)	< 0.001	4.95 (3.22–7.60)	< 0.001
Multiple cancer surgeries	30.40 (16.97–54.44)	< 0.001	11.86 (6.54–21.50)	< 0.001
Income level at the index procedure				
1st quartile (lowest)	Reference		Reference	
2nd quartile	0.57 (0.49–0.66)	< 0.001	0.70 (0.60–0.82)	< 0.001
3rd quartile	0.64 (0.55–0.74)	< 0.001	0.70 (0.60–0.82)	< 0.001
4th quartile (highest)	0.82 (0.71–0.94)	0.006	0.77 (0.67–0.90)	0.001
Residence level at the index procedure				
Capital city	Reference		Reference	
Metropolitan city	1.05 (0.89–1.25)	0.571	1.16 (0.97–1.38)	0.098
Other area	1.16 (1.00–1.34)	0.050	1.02 (0.88–1.18)	0.840

*COVID-19*, coronavirus disease 2019; *OR*, odds ratio; *CI*, confidence interval

Exploratory subgroup analyses (Supplemental Tables 3 and 4) revealed that only the cancer surgery type significantly impacted the association between preoperative COVID-19 and 30-day postoperative mortality (*P* < 0.001). No significant differences were observed in the primary outcome across the other subgroups. The results of the logistic regression analyses on the primary and secondary outcomes of abdominopelvic cancer surgery are provided in Supplemental Tables 5 and 6.

## Discussion

This study identified a significant association between COVID-19 diagnosed within 2 weeks before surgery and increased 30-day and 90-day mortality rates after elective cancer surgery. This finding corroborates recent guidelines that suggest postponing elective surgery at least 2 weeks after COVID-19 is diagnosed.^4,5^ Furthermore, preoperative administration of the COVID-19 vaccine was significantly associated with reduced 30-day and 90-day postoperative mortality rates, thereby underscoring the importance of vaccination for patients who require surgery.

Recent studies have suggested shortening the time between elective surgeries and the COVID-19 diagnosis from 7 to 2 weeks.^23–25^ Additionally, a study performed in the United Kingdom reported a 1.1% postoperative mortality rate for surgeries within 2 weeks of COVID-19 infection, decreasing to 0.3% between 4 and 6 weeks, and aligning with the mortality rate in COVID-19 patients beyond 6 weeks or no preoperative COVID-19 infection.^24^ However, that study did not adjust for variables that could affect mortality and included various surgeries other than cancer surgeries. A multicenter study performed in France during the Omicron-predominant postvaccine era did not find significant associations between COVID-19 diagnosed within 3 weeks before surgery and increased postoperative respiratory morbidities.^23^ However, that study included only 4928 patients, and 705 of those patients were diagnosed with COVID-19 within 8 weeks before surgery; therefore, it may have been underpowered to detect the influence of preoperative COVID-19 on postoperative outcomes.^23^ Despite these limitations, that study emphasized the significance of its findings by considering that delaying time-sensitive surgeries could worsen the postoperative prognosis.^23^ Our study offers new insights regarding the timing of cancer surgeries after the diagnosis of COVID-19, thereby adding beneficial information to the literature.

The results of our current and previous studies reflect recent changes in the impact of COVID-19 on postoperative outcomes.^26^ Although our previous study included a variety of surgeries, including cancer surgeries, the 30-day and 90-day postoperative mortality rates among patients without preoperative COVID-19 were similar to those observed during the current study.^26^ However, our previous study, which focused solely on surgeries conducted in 2021, found that COVID-19 diagnosed up to 8 weeks before surgery was associated with a postoperative mortality rate that was more than double that of patients without preoperative COVID-19 (1.4% vs. 0.4%).^26^ Furthermore, the adjusted OR for 30-day postoperative mortality observed during our previous study was higher than that observed during our current study (adjusted ORs 0–4 weeks, 4.28 [95% CI 1.81–10.31; *P* = 0.001]; 4–8 weeks, 3.38 [95% CI 1.54–7.44; *P* = 0.002]). Because the patients included in this study underwent surgery in 2022, the observed differences were likely attributable to the overall weakened impact of preoperative COVID-19 on surgical patients. According to recent data from the KDCA, compared with the severity and fatality rates of COVID-19 during the pre-delta and delta-dominant periods, those of the Omicron-dominant period, which began in January 2022, significantly decreased.^27^

Our study found a significant association between elective cancer surgeries conducted within 2 weeks of the COVID-19 diagnosis and increased postoperative mortality; however, no such association was observed for surgeries performed thereafter. This finding suggests the need to delay cancer surgeries for at least 2 weeks for patients with preoperative COVID-19 in agreement with recent guidelines.^4,5^ Fortunately, this delay falls within the 3-week “safe postponement period” for patients who require cancer surgery, as suggested by a recent study performed in the United States that used the National Cancer Database.^28^ In addition, the evaluation of robustness using E-values revealed that the nonsignificant association between surgeries performed more than 2 weeks after COVID-19 infection and 30-day postoperative mortality is more robust than the significant association found with surgeries conducted within 2 weeks of a COVID-19 infection. This finding underscores that delaying cancer surgeries for more than 2 weeks because of COVID-19 infection may not be necessary. Therefore, postponing cancer surgery for 2 weeks because of COVID-19 is likely to be an appropriate decision, and there is no justification for further delays.

Our study also found that being fully vaccinated against COVID-19 before surgery reduced early postoperative mortality. A retrospective study conducted in the United States found that the preoperative COVID-19 vaccination status could influence perioperative complications.^19^ Among a fully vaccinated cohort, COVID-19 diagnosed within 4 weeks before surgery was not significantly associated with increased perioperative complications. However, among the cohort that was not fully vaccinated, COVID-19 diagnosed within 4 weeks before surgery was significantly associated with increased perioperative complications.^19^ Other retrospective studies performed in the United States showed that being fully vaccinated against COVID-19 decreased perioperative complications and postoperative mortality.^20,29^ However, our previous study did not show an association between being fully vaccinated and 30-day postoperative mortality,^24^ possibly because vaccinations were initiated later in South Korea than in other countries and administered to older adults first because they were at higher risk.^30^ In South Korea, vaccinations became available for all adults on August 26, 2021; therefore, most patients who had received a second preoperative COVID-19 vaccination were likely to be older in our previous study.^26^ The unadjusted OR for preoperative COVID-19 vaccination in our previous study was associated with an increased risk of 30-day postoperative mortality.^26^ However, our current findings did not show this increased risk because vaccinations were available for all adults.

This study had a few limitations. First, because of the retrospective design of this study, unmeasured confounders that could affect the primary and secondary outcomes—such as the extent of the surgery, the severity of the patient’s underlying conditions, and the experience of the medical institution—could have influenced the results. As previously mentioned, the robustness of the significant association between COVID-19 infections occurring within 2 weeks preoperatively and an increase in the 30-day mortality, as assessed using the E value, was not substantial. Therefore, our study results should be interpreted cautiously. Second, it focused solely on cancer surgeries performed in South Korea. Because of global variations in cancer treatment outcomes, this limited the generalizability of our findings.^31^ However, South Korea provides excellent cancer treatment,^32^ which likely minimized the impact of surgical quality on our study results. Third, the postponement of cancer surgery can be influenced not only by individual medical reasons but also by the healthcare environment and socioeconomic factors.^33^ We were unable to obtain information regarding the decision-making process in terms of surgical timing for patients with preoperative COVID-19. Fourth, our reliance on national health insurance data restricted our ability to assess the severity of preoperative COVID-19. According to a study that utilized data from the United States National COVID Cohort Collaborative, the severity of COVID-19 could influence the association between the duration of the COVID-19 diagnosis and surgery and the occurrence of major adverse cardiovascular and cerebrovascular events within 30 days postoperatively.^25^ However, according to the intermediate results of a chronic COVID-19 syndrome study that was recently published by the Korea National Institute of Health, only 0.4% of patients with COVID-19 in South Korea were diagnosed with post-COVID-19 conditions.^34^ Therefore, the proportion of such patients was very low, leading us to cautiously conclude that this did not significantly impact our study results. Fifth, the long-term postoperative effects of COVID-19 were not assessed, and data beyond 90 days after surgery were lacking. Because COVID-19 is constantly evolving, we included the most recent patient cohort up to December 2022 to observe contemporary trends. During a study that examined the impact of preoperative COVID-19 on mid-term postoperative outcomes, a significant divergence in the prognosis based on the presence of COVID-19 was observed soon after surgery.^35^ Consequently, the results of our study highlight the risks posed by preoperative COVID-19 to the postoperative prognosis. Sixth, interactions between the cancer surgery type and postoperative mortality were not fully analyzed, because only a few such events occurred. We anticipate a more pronounced association with surgeries with greater severity than with those with lower postoperative mortality rates, such as thyroid surgeries. Finally, resource constraints during the pandemic might have affected the study.^36^ However, the efficient response of South Korea may have mitigated these effects.^37–39^

Although our results support delaying cancer surgeries for at least 2 weeks after COVID-19 is diagnosed, they did not conclusively establish the safety of conducting surgeries beyond 2 weeks after infection. Factors, such as the severity of COVID-19 and related symptoms, should be considered. However, delaying cancer surgeries for at least 2 weeks for patients with COVID-19 is a reasonable strategy to mitigate the risk of worsened prognoses caused by infection. Importantly, this timeframe is unlikely to negatively affect cancer surgery outcomes, thus making it a feasible and practical approach.

## Supplementary Information

Below is the link to the electronic supplementary material.

Supplementary file1 (DOCX 46 kb)
