# Supplementary material for: Preoperative COVID-19 and Postoperative Mortality in Cancer Surgery: A South Korean Nationwide Study
Source: Ann Surg Oncol. 2024 Jun 15;31(10):6394–404. doi: 10.1245/s10434-024-15594-1 (PMC11413177; doi:10.1245/s10434-024-15594-1)
Supplement: Supplementary file 1 — Supplementary file1 (DOCX 46 kb) [file 10434_2024_15594_MOESM1_ESM.docx]

**Supplementary Online Content**

**Preoperative COVID-19 and Postoperative Mortality in Cancer Surgery: A South Korean Nationwide Study**

Jae-Woo Ju, MD^1,2^, Soo-Hyuk Yoon, MD^1^, Tak Kyu Oh, MD, PhD^2,3^, and Ho-Jin Lee, MD, PhD^1,2,*^

^1^ Department of Anesthesiology and Pain Medicine, Seoul National University Hospital, Seoul, Republic of Korea

^2^ Department of Anesthesiology and Pain Medicine, Seoul National University College of Medicine, Seoul, Republic of Korea

^3^ Department of Anesthesiology and Pain Medicine, Seoul National University Bundang Hospital, Seongnam, Republic of Korea

**^*^****Corresponding Author:** Ho-Jin Lee, MD, PhD

Address: Department of Anesthesiology and Pain Medicine, Seoul National University Hospital, 101 Daehak-ro, Jongno-gu, Seoul 03080, Republic of Korea

Phone: 82-2-2072-0039

Fax: 82-2-747-8363

E-mail: [hjpainfree@snu.ac.kr](mailto:hjpainfree@snu.ac.kr)

**TABLE OF CONTENTS**

**Supplemental Table 1.** The ICD-10 and procedure codes for the cancer surgery … p. 3

**Supplemental Table 2.** The ICD-10 codes for the covariates … p. 4

**Supplemental Table 3.** Incidence of 30-day postoperative mortality after elective cancer surgery in each subgroup … p. 5

**Supplemental Table 4.** Subgroup analyses of 30-day postoperative mortality after elective cancer surgery … p. 7

**Supplemental Table 5.** Univariable and multivariable logistic regression analyses for 30-day postoperative mortality after elective abdominopelvic cancer surgery… p. 9

**Supplemental Table 6.** Univariable and multivariable logistic regression analyses for 90-day postoperative mortality after elective abdominopelvic cancer surgery… p. 10

**Supplemental Table 1.** The ICD-10 and procedure codes for the cancer surgery

| **Type of cancer surgery** | **ICD-10 codes** | **Procedure codes** |
| --- | --- | --- |
| Thyroid | C73, D0930 | P4551, P4552, P4553, P4554, P4561, P4543 |
| Breast | C50, D05 | N7131, N7132, N7133, N7134, N7135, N7136, N7137, N7138, N7139 |
| Stomach | C16, D002 | QA536, Q2533, Q2534, Q2536, Q2537, Q2550, Q2551, Q2552, Q2561, Q2562, Q2571, Q2572, Q2573, Q0259, Q2594, Q2598, Q0251, Q0252, Q0253, Q0258, Q0256, Q0257, Q0255, Q0254 |
| Lung | C34, D022 | O1401, O1402, O1403, O1404, O1405, O1410, O1421, O1422, O1423, O1424, O1431, O1432, O1450, O1471 |
| Liver | C22, D015 | Q7221, Q7222, Q7223, Q7224, Q7225, Q8040, Q8041, Q8042, Q8043, Q8044, Q8045, Q8046, Q8047, Q8048, Q8049, Q8050, Q8140, Q8141, Q8142, Q8143, Q8144, Q8145, Q8146, Q8147, Q8148, Q8149, Q8150, Q7280, Q7281, Q7284, Q7285, Q7282, Q7283, Q7230, M6644 |
| Gallbladder | C23, D0151 | Q7410, Q7380 |
| Pancreas | C25, D017 | Q7230, Q7561, Q7562, Q7563, Q7564, Q7565, Q7566, Q7567, Q7571, Q7572, Q7352, Q2572, Q2573, Q2680 |
| Colorectal | C18, C19, C20, D010, D011 | QA671, QA672, QA673, QA679, Q2671, Q2672, Q2673, Q2679, Q1261, Q1262, QA921, QA922, QA923, QA924, Q2921, Q2922, Q2923, Q2924, Q2927, Q0292, QA928, Q2928, QA925, QA926, Q2925, Q2926, Q2791, Q2792, Q2793, Q2794, Q2796, Q2797, Q2798, Q2680, Q2891, Q2890, Q2892, Q2893, R4156 |
| Uterus | C53, C54, D06, D070 | R4250, R4154, R4155, R4156, R4157, R4143, R4144, R4145, R4146 |
| Ovary | C56 | R4423, R4424, R4427, R4428, R4157, R4425, R4426 |
| Kidney | C64 | M6890, R3273, R3290, R3305, R3307, R3308 |
| Prostate | C61, D075 | RZ512, R3960, R3976 |
| Testis | C62, D076 | R3851, R3852, R3853, R3861, R3862, R3871, R3881, R3883, R3882, R3891 |

ICD-10, International Classification of Diseases, tenth revision

**Supplemental Table 2.** The ICD-10 codes for the covariates

|  | **ICD-10 codes** | **Updated weight** |
| --- | --- | --- |
| Congestive heart failure | I09.9, I11.0, I13.0, I13.2, I25.5, I42.0, I42.5 - I42.9, I43.x, I50.x, P29.0 | 2 |
| Dementia | F00.x - F03.x, F05.1, G30.x, G31.1 | 2 |
| Chronic pulmonary disease | I27.8, I27.9, J40.x - J47.x, J60.x - J67.x, J68.4, J70.1, J70.3 | 1 |
| Rheumatic disease | M05.x, M06.x, M31.5, M32.x - M34.x, M35.1, M35.3, M36.0 | 1 |
| Mild liver disease | B18.x, K70.0 - K70.3, K70.9, K71.3 - K71.5, K71.7, K73.x, K74.x, K76.0, K76.2 - K76.4, K76.8, K76.9, Z94.4 | 2 |
| Diabetes with chronic complication | E10.2 - E10.5, E10.7, E11.2 - E11.5, E11.7, E12.2 - E12.5, E12.7, E13.2 - E13.5, E13.7, E14.2 - E14.5, E14.7 | 1 |
| Hemiplegia or paraplegia | G04.1, G11.4, G80.1, G80.2, G81.x, G82.x, G83.0 - G83.4, G83.9 | 2 |
| Renal disease | I12.0, I13.1, N03.2 - N03.7, N05.2 - N05.7, N18.x, N19.x, N25.0, Z49.0 - Z49.2, Z94.0, Z99.2 | 1 |
| Any malignancy, including lymphoma and leukemia | C00.x - C26.x, C30.x - C34.x, C37.x - C41.x, C43.x, C45.x - C58.x, C60.x - C76.x, C81.x - C85.x, C88.x, C90.x - C97.x | 2 |
| Moderate or severe liver disease | I85.0, I85.9, I86.4, I98.2, K70.4, K71.1, K72.1, K72.9, K76.5, K76.6, K76.7 | 4 |
| Metastatic solid tumor | C77.x - C80.x | 6 |
| AIDS/HIV | B20.x - B22.x, B24.x | 4 |

AIDS, acquired immune deficiency syndrome; HIV, human immunodeficiency virus; ICD-10, International Classification of Diseases, tenth revision

**Supplemental Table 3.** Incidence of 30-day postoperative mortality after elective cancer surgery in each subgroup

|  | **No preoperative COVID-19**  **(n = 68,622)** | **Preoperative COVID-19 infection**  **(by timing of diagnosis prior to surgery)** | | | |
| --- | --- | --- | --- | --- | --- |
|  |  | **0–2 weeks**  **(n = 3,489)** | **3-4 weeks**  **(n = 2,841)** | **5-6 weeks**  **(n = 2,270)** | **≥7 weeks**  **(n = 22,333)** |
| Age, years |  |  |  |  |  |
| 19–49 | 10/16,114 (0.1) | 1/763 (0.1) | 0/812 (0.0) | 2/700 (0.3) | 3/7,630 (0.0) |
| 50–69 | 95/35,161 (0.3) | 11/1,675 (0.7) | 1/1,334 (0.1) | 4/1,046 (0.4) | 18/10,177 (0.2) |
| ≥ 70 | 221/17,347 (1.3) | 20/1,051 (1.9) | 11/695 (1.6) | 8/524 (1.5) | 44/4,526 (1.0) |
| Sex |  |  |  |  |  |
| Female | 117/43,314 (0.3) | 3/2,152 (0.1) | 3/1,925 (0.2) | 5/1,524 (0.3) | 21/15,781 (0.1) |
| Male | 209/25,308 (0.8) | 29/1,337 (2.2) | 9/916 (1.0) | 9/746 (1.2) | 44/6,552 (0.7) |
| Updated Charlson comorbidity index score |  |  |  |  |  |
| 0–2 | 23/17,850 (0.1) | 1/646 (0.2) | 0/603 (0.0) | 3/503 (0.6) | 2/5,021 (0.0) |
| 3-4 | 51/19,401 (0.3) | 9/983 (0.9) | 1/810 (0.1) | 0/658 (0.0) | 7/6,573 (0.1) |
| ≥ 5 | 252/31,371 (0.8) | 22/1,860 (1.2) | 11/1,428 (0.8) | 11/1,109 (1.0) | 56/10,739 (0.5) |
| Vaccination |  |  |  |  |  |
| Not or not fully vaccinated | 51/4928 (1.0) | 5/258 (1.9) | 1/200 (0.5) | 1/167 (0.6) | 8/1,351 (0.6) |
| Fully vaccinated | 275/63,694 (0.4) | 27/3,231 (0.8) | 11/2,641 (0.4) | 13/2,103 (0.6) | 57/20,982 (0.3) |
| Type of cancer surgery |  |  |  |  |  |
| Thyroid | 9/14,171 (0.1) | 1/582 (0.2) | 0/654 (0.0) | 0/623 (0.0) | 1/6,023 (0.0) |
| Breast | 6/17,465 (0.0) | 0/867 (0.0) | 0/780 (0.0) | 0/532 (0.0) | 1/6,398 (0.0) |
| Stomach | 44/8,318 (0.5) | 9/428 (2.1) | 3/293 (1.0) | 0/202 (0.0) | 12/1,970 (0.6) |
| Colorectal | 156/11,778 (1.3) | 15/824 (1.8) | 4/435 (0.9) | 6/316 (1.9) | 29/2,665 (1.1) |
| Hepatobiliary | 53/4,545 (1.2) | 3/251 (1.2) | 3/163 (1.8) | 3/138 (2.2) | 11/1,123 (1.0) |
| Genitourinary | 19/5,523 (0.3) | 3/211 (1.4) | 0/202 (0.0) | 0/192 (0.0) | 3/1,900 (0.2) |
| Lung | 37/6,417 (0.6) | 1/297 (0.3) | 1/291 (0.3) | 5/250 (2.0) | 6/2,148 (0.3) |
| Multiple cancer surgeries | 2/405 (0.5) | 0/29 (0.0) | 1/23 (4.3) | 0/17 (0.0) | 2/106 (1.9) |
| Income level at the index procedure |  |  |  |  |  |
| First quartile (lowest) | 134/19,286 (0.7) | 7/989 (0.7) | 4/827 (0.5) | 3/641 (0.5) | 29/6,248 (0.5) |
| Second quartile | 56/18,566 (0.3) | 6/947 (0.6) | 1/751 (0.1) | 3/638 (0.5) | 9/6,454 (0.1) |
| Third quartile | 72/15,299 (0.5) | 10/772 (1.3) | 2/624 (0.3) | 3/479 (0.6) | 12/4,793 (0.3) |
| Fourth quartile (highest) | 64/15,471 (0.4) | 9/781 (1.2) | 5/639 (0.8) | 5/512 (1.0) | 15/4,838 (0.3) |
| Area of residence at the index procedure |  |  |  |  |  |
| Capital city | 58/13,001 (0.4) | 1/620 (0.2) | 2/600 (0.3) | 4/464 (0.9) | 12/4,768 (0.3) |
| Metropolitan city | 71/17,076 (0.4) | 4/738 (0.5) | 1/725 (0.1) | 4/540 (0.7) | 11/5,748 (0.2) |
| Other areas | 197/38,545 (0.5) | 27/2,131 (1.3) | 9/1,516 (0.6) | 6/1,266 (0.5) | 42/11,817 (0.4) |

COVID-19, Coronavirus disease 2019

**Supplemental Table 4.** Subgroup analyses of 30-day postoperative mortality after elective cancer surgery

|  | **Adjusted odds ratio (95% confidence interval)**^*^ | | | | |
| --- | --- | --- | --- | --- | --- |
|  | **COVID–19 infection**  **0–2 weeks prior to surgery** | **COVID-19 infection**  **3–4 weeks prior to surgery** | **COVID-19 infection**  **5–6 weeks prior to surgery** | **COVID-19 infection**  **≥7 weeks prior to surgery** | **P-value for**  **interaction** |
| Age, years |  |  |  |  | 0.532 |
| 19–49 | 2.57 (0.47–14.11) | 1.07 (0.06–17.80) | 6.30 (1.60–24.87) | 0.90 (0.27–2.95) |  |
| 50–69 | 2.07 (1.12–3.83) | 0.46 (0.09–2.26) | 1.61 (0.63–4.15) | 0.79 (0.48–1.30) |  |
| ≥ 70 | 1.27 (0.81–2.01) | 1.14 (0.63–2.08) | 1.30 (0.65–2.58) | 0.77 (0.56–1.07) |  |
| Sex |  |  |  |  | 0.058 |
| Female | 0.43 (0.15–1.24) | 0.65 (0.23–1.88) | 1.38 (0.59–3.26) | 0.62 (0.39–0.98) |  |
| Male | 2.11 (1.43–3.13) | 1.12 (0.58–2.15) | 1.64 (0.85–3.16) | 0.88 (0.64–1.22) |  |
| Updated Charlson comorbidity index score |  |  |  |  | 0.066 |
| 0–2 | 1.48 (0.28–7.72) | 0.82 (0.05–13.30) | 6.54 (2.11–20.27) | 0.60 (0.17–2.20) |  |
| 3-4 | 3.55 (1.77–7.13) | 0.90 (0.18–4.53) | 0.39 (0.03–6.23) | 0.64 (0.30–1.38) |  |
| ≥ 5 | 1.21 (0.78–1.87) | 0.99 (0.55–1.80) | 1.45 (0.80–2.63) | 0.82 (0.61–1.09) |  |
| Vaccination |  |  |  |  | 0.945 |
| Not or not fully vaccinated | 1.29 (0.52–3.18) | 0.61 (0.12–3.16) | 1.03 (0.20–5.38) | 0.68 (0.33–1.41) |  |
| Fully vaccinated | 1.53 (1.03–2.27) | 1.01 (0.56–1.81) | 1.63 (0.94–2.83) | 0.80 (0.60–1.06) |  |
| Type of cancer surgery |  |  |  |  | <0.001 |
| Thyroid | 1.89 (1.88–1.90) | 1.01 (1.00–1.02) | 1.16 (1.15–1.17) | 0.76 (0.75–0.76) |  |
| Breast | 1.05 (1.00–1.10) | 1.04 (0.99–1.09) | 1.26 (1.19–1.33) | 0.82 (0.80–0.83) |  |
| Stomach | 6.88 (6.79–6.97) | 2.08 (2.03–2.14) | 0.51 (0.48–0.54) | 1.16 (1.15–1.18) |  |
| Colorectal | 2.15 (2.13–2.18) | 0.73 (0.72–0.75) | 2.84 (2.79–2.88) | 0.88 (0.87–0.89) |  |
| Hepatobiliary | 1.28 (1.25–1.31) | 3.32 (3.25–3.40) | 4.66 (4.58–4.76) | 0.90 (0.89–0.92) |  |
| Genitourinary | 5.59 (5.46–5.73) | 0.78 (0.73–0.83) | 0.78 (0.73–0.83) | 0.61 (0.59–0.62) |  |
| Lung | 0.81 (0.78–0.84) | 0.91 (0.87–0.94) | 6.32 (6.20–6.43) | 0.55 (0.54–0.56) |  |
| Multiple cancer surgeries | 2.97 (2.80–3.15) | –inf (–inf to inf) | 8.03 (7.68–8.39) | 6.60 (6.45–6.76) |  |
| Income level at the index procedure |  |  |  |  | 0.582 |
| First quartile (lowest) | 0.85 (0.40–1.77) | 0.79 (0.31–2.03) | 0.89 (0.31–2.59) | 0.85 (0.57–1.26) |  |
| Second quartile | 1.71 (0.76–3.88) | 0.63 (0.12–3.18) | 1.81 (0.61–5.38) | 0.64 (0.32–1.27) |  |
| Third quartile | 2.07 (1.07–4.00) | 0.76 (0.21–2.69) | 1.87 (0.63–5.54) | 0.69 (0.38–1.26) |  |
| Fourth quartile (highest) | 2.10 (1.05–4.20) | 2.15 (0.89–5.19) | 2.69 (1.11–6.51) | 0.90 (0.52–1.57) |  |
| Area of residence at the index procedure |  |  |  |  | 0.455 |
| Capital city | 0.36 (0.07–1.82) | 0.88 (0.25–3.14) | 2.48 (0.93–6.57) | 0.70 (0.38–1.28) |  |
| Metropolitan city | 1.23 (0.47–3.19) | 0.56 (0.11–2.79) | 2.21 (0.84–5.81) | 0.67 (0.36–1.25) |  |
| Other areas | 1.93 (1.29–2.89) | 1.14 (0.59–2.20) | 1.10 (0.50–2.42) | 0.85 (0.61–1.18) |  |

^*^Referenced to the patients without preoperative COVID-19 infection

COVID-19, Coronavirus disease 2019

**Supplemental Table 5.** Univariable and multivariable logistic regression analyses for 30-day postoperative mortality after elective abdominopelvic cancer surgery

|  | Univariable | | Multivariable | |
| --- | --- | --- | --- | --- |
|  | Unadjusted OR  (95% CI) | P-value | Adjusted OR  (95% CI) | P-value |
| Timing of diagnosis of COVID-19 prior to surgery |  |  |  |  |
| No preoperative COVID-19 | Reference |  | Reference |  |
| 0–2 weeks | 1.99 (1.36–2.90) | < 0.001 | 1.69 (1.16–2.47) | 0.007 |
| 3–4 weeks | 1.06 (0.57–1.98) | 0.847 | 0.94 (0.50–1.74) | 0.838 |
| 5–6 weeks | 1.24 (0.65–2.38) | 0.515 | 1.26 (0.66–2.41) | 0.487 |
| ≥7 weeks | 0.80 (0.60–1.07) | 0.133 | 0.82 (0.61–1.09) | 0.165 |
| Age, years |  |  |  |  |
| 0–49 | Reference |  | Reference |  |
| 50–69 | 1.64 (0.96–2.79) | 0.070 | 1.43 (0.84–2.44) | 0.187 |
| ≥70 | 5.22 (3.13–8.73) | < 0.001 | 4.39 (2.61– 7.37) | < 0.001 |
| Male (versus female) | 1.48 (1.20–1.83) | < 0.001 | 1.45 (1.17–1.79) | < 0.001 |
| Updated Charlson comorbidity index |  |  |  |  |
| 0-3 | Reference |  | Reference |  |
| 4-5 | 1.44 (0.92–2.26) | 0.116 | 1.26 (0.80–1.97) | 0.320 |
| ≥6 | 3.69 (2.49–5.46) | < 0.001 | 2.59 (1.75–3.83) | < 0.001 |
| Fully vaccinated (versus not or not fully vaccinated) | 0.36 (0.27–0.47) | < 0.001 | 0.32 (0.24–0.42) | < 0.001 |
| Income level at the index procedure |  |  |  |  |
| 1^st^ quartile (lowest) | Reference |  | Reference |  |
| 2^nd^ quartile | 0.45 (0.33–0.61) | < 0.001 | 0.51 (0.38–0.69) | < 0.001 |
| 3^rd^ quartile | 0.76 (0.58–1.00) | 0.046 | 0.79 (0.60–1.03) | 0.079 |
| 4^th^ quartile (highest) | 0.74 (0.57–0.97) | 0.029 | 0.64 (0.49–0.84) | 0.001 |
| Residence level at the index procedure |  |  |  |  |
| Capital city | Reference |  | Reference |  |
| Metropolitan city | 0.95 (0.68–1.33) | 0.773 | 1.03 (0.74–1.43) | 0.875 |
| Other area | 1.22 (0.92–1.61) | 0.160 | 1.14 (0.86–1.50) | 0.370 |

COVID-19, Coronavirus disease 2019; OR, odds ratio; CI, confidence interval.

**Supplemental Table 6.** Univariable and multivariable logistic regression analyses for 90-day postoperative mortality after elective abdominopelvic cancer surgery

|  | Univariable | | Multivariable | |
| --- | --- | --- | --- | --- |
|  | Unadjusted OR  (95% CI) | P-value | Adjusted OR  (95% CI) | P-value |
| Timing of diagnosis of COVID-19 prior to surgery |  |  |  |  |
| No preoperative COVID-19 | Reference |  | Reference |  |
| 0–2 weeks | 2.00 (1.60–2.52) | < 0.001 | 1.73 (1.37–2.18) | < 0.001 |
| 3–4 weeks | 1.30 (0.93–1.82) | 0.127 | 1.15 (0.82–1.63) | 0.414 |
| 5–6 weeks | 1.31 (0.90–1.92) | 0.165 | 1.29 (0.88–1.89) | 0.202 |
| ≥7 weeks | 0.93 (0.79–1.10) | 0.385 | 0.93 (0.79–1.09) | 0.376 |
| Age, years |  |  |  |  |
| 0–49 | Reference |  | Reference |  |
| 50–69 | 1.71 (1.27–2.32) | < 0.001 | 1.50 (1.10–2.03) | < 0.001 |
| ≥70 | 4.81 (3.59–6.44) | < 0.001 | 3.90 (2.89– 5.26) | < 0.001 |
| Male (versus female) | 1.36 (1.20–1.53) | < 0.001 | 1.34 (1.18–1.52) | < 0.001 |
| Updated Charlson comorbidity index |  |  |  |  |
| 0-3 | Reference |  | Reference |  |
| 4-5 | 1.12 (0.85–1.49) | 0.422 | 1.00 (0.75–1.32) | 0.985 |
| ≥6 | 4.11 (3.26–5.18) | < 0.001 | 3.04 (2.40–3.84) | < 0.001 |
| Fully vaccinated (versus not or not fully vaccinated) | 0.37 (0.32–0.44) | < 0.001 | 0.34 (0.28–0.40) | < 0.001 |
| Income level at the index procedure |  |  |  |  |
| 1^st^ quartile (lowest) | Reference |  | Reference |  |
| 2^nd^ quartile | 0.58 (0.49–0.69) | < 0.001 | 0.65 (0.55–0.77) | < 0.001 |
| 3^rd^ quartile | 0.66 (0.56–0.78) | < 0.001 | 0.69 (0.58–0.82) | < 0.001 |
| 4^th^ quartile (highest) | 0.85 (0.73–1.00) | 0.047 | 0.76 (0.65–0.89) | < 0.001 |
| Residence level at the index procedure |  |  |  |  |
| Capital city | Reference |  | Reference |  |
| Metropolitan city | 1.08 (0.90–1.30) | 0.413 | 1.17 (0.97–1.41) | 0.100 |
| Other area | 1.07 (0.91–1.26) | 0.413 | 1.00 (0.85–1.17) | 0.962 |

COVID-19, Coronavirus disease 2019; OR, odds ratio; CI, confidence interval.
